# The societal impact of Open Science: a scoping review

**DOI:** 10.1098/rsos.240286

**Published:** 2024-06-27

**Authors:** Nicki Lisa Cole, Eva Kormann, Thomas Klebel, Simon Apartis, Tony Ross-Hellauer

**Affiliations:** Open and Reproducible Research Group, Sandgasse 36, 8010 Graz, Austria

**Keywords:** societal impact, social impact, open science, citizen science, participatory research, open access

## Abstract

Open Science (OS) aims, in part, to drive greater societal impact of academic research. Government, funder and institutional policies state that it should further democratize research and increase learning and awareness, evidence-based policy-making, the relevance of research to society's problems, and public trust in research. Yet, measuring the societal impact of OS has proven challenging and synthesized evidence of it is lacking. This study fills this gap by systematically scoping the existing evidence of societal impact driven by OS and its various aspects, including Citizen Science (CS), Open Access (OA), Open/FAIR Data (OFD), Open Code/Software and others. Using the PRISMA Extension for Scoping Reviews and searches conducted in Web of Science, Scopus and relevant grey literature, we identified 196 studies that contain evidence of societal impact. The majority concern CS, with some focused on OA, and only a few addressing other aspects. Key areas of impact found are education and awareness, climate and environment, and social engagement. We found no literature documenting evidence of the societal impact of OFD and limited evidence of societal impact in terms of policy, health, and trust in academic research. Our findings demonstrate a critical need for additional evidence and suggest practical and policy implications.

## 1. Introduction

Recent decades have seen increased efforts, on the part of research funders, institutions and governmental organizations to foster, monitor and demonstrate the impacts of funded research beyond the academy [1–3]. Within Europe, both the United Kingdom (UK) [4] and The Netherlands assess the societal impact of research alongside other quality criteria [1], and the European Commission (EC) has placed considerable focus on societal impact in recent funding Framework Programmes [1]. The Horizon Europe (HE) Framework Programme includes a focus on ensuring that funded research addresses European Union (EU) policy priorities and global challenges, delivers benefits and impact through research and innovation missions, and strengthens the uptake of research and innovation in society [5]. Similar policy approaches are taken in numerous other countries [6]. For example, Austria aims to foster the societal impact of research through its ‘open innovation’ policy and funding for Citizen Science and participatory research, while The Netherlands offers funding for societal engagement in research through participatory and community-based models [6]. Beyond Europe, societal impact of research is monitored by the Australian government [1], and in the USA, federal legislation urges higher education institutions (HEIs) and funders to create ‘broader impacts’ of research, including ‘societal benefits’ [7] and the US Office of Science and Technology Policy (OSTP) includes a dedicated Science and Society team that aims to ‘ensur[e] all of America can participate in, contribute to, and benefit from science and technology’ [8]. Science policy in India aims to drive societal impact with the encouragement of science–society connections in a variety of ways, including funding for community-based research [6].

The adoption of Open Science (OS) policies by research institutions and funders globally aligns with the aim of fostering societal impact. OS is both a set of practices and a research reform movement that aims to make academic research (henceforth, ‘research’) more transparent, inclusive and accessible [9]. It includes diverse practices like Open Access (OA) to research publications and Open and FAIR Data (OFD); the creation and use of Open Code and Software; practising process transparency through pre-registration (specifying a research plan in advance and publishing it in a publicly accessible registry (see [10])) and Open Methods; evaluation transparency through Open Evaluation (e.g. Open Peer Review); and Citizen Science (CS), co-creation, participatory research and collaboration [11,12]. Collectively, through these practices, OS aims to make research and the knowledge it generates freely accessible and useful outside of academia, to make research processes more collaborative and efficient, to create an open infrastructure system to support and enable open practices and free access, and to create new ways of assessing the value of research which break with traditional metrics [13].

Implicit in these aims is the belief that OS can yield greater societal impact as compared with ‘closed’ research. UNESCO's definition of OS includes the concept of benefitting society, and in its Recommendation on Open Science, it asserts that increasing openness should ‘enhanc[e] the social impact of science and increas[e] the capacity of society as a whole to solve complex interconnected problems' [11]. The European Commission's (EC) policy on OS includes the assertion that OS makes research ‘more responsive to society's needs’ [14]. This open approach to research and innovation is intended to best support the pursuit of solutions to ‘societal challenges’, or in other words, achieve maximum societal benefit. Yet, to date, there is limited evidence as to whether OS policies and practices are achieving this goal.

While ‘research impact’ is a vague concept that lacks a coherent and consistent definition [15], some attempts have been made to define societal impact through literature reviews on the topic [1,4]. As stated in a literature review from the Ludwig Boltzmann Gesellschaft (LBG), ‘Societal impact […] focuses on the effects and changes that research activities unfold beyond academia in other areas of life such as society, culture, public services, health or the environment’, and can include changes in practice, policy and legislation, and to awareness, understanding and individual knowledge and skills [1]. A review by Bornmann does not result in a concise definition but lists similar aspects to the LBG review and includes economic impacts [4]. Critical to the LBG definition of societal impact is that it is demonstrable; e.g. there is evidence that research outputs are used in policy-making or that they inform the improvement of healthcare delivery, among others. Adding nuance, the authors assert that societal impact may be instrumental, like the examples listed in the prior sentence, conceptual (e.g. changes to awareness, understanding or perspective), or attitudinal or cultural (e.g. behavioural changes). It can also take the form of capacity building, such as long-term impacts that manifest through knowledge, skills gain or the development of relationships between diverse stakeholders [1]. Additionally, Bornmann notes that societal impact ‘is not a short-term phenomenon’, but rather ‘only becomes apparent in the distant future’ [4]. Other ‘hallmarks’ of it include that it can be either anticipated or unanticipated, within or outside of the intended area, geographically limited or global [4].

Extending this prior work within the PathOS project^1^, for which this review was conducted, we define impact (generally) as ‘long-lasting, elementary and wide-spread change’ and understand that it can be ‘direct or indirect, intended or unintended, [and] relate to behavioural and/or systemic changes’ [16]. Further, we conceptualize *societal impact* as a composite of multiple things. This includes and is not limited to (1) social impact (contribution to community welfare, quality of life, behaviour, practices and activities of and relationships among and between people and groups), (2) cultural impact (contribution to understanding of ideas and reality, values and beliefs), (3) political impact (contribution to how policy makers act and how policies are constructed, and to governance and administration of society), (4) environmental impact (contribution to the management of the environment, for example, natural resources, environmental pollution, climate and meteorology), and (5) health impact (contribution to public health, life expectancy, prevention of illnesses and disease, community safety) [16]. We consider economic impact of OS separate from societal impact, and note that our colleagues investigated it, and academic impact, through separate scoping reviews [17].

We recognize that measuring societal impact is difficult, primarily because of challenges related to causality, but also due to a host of other issues [2]. For example, as Wehn *et al*. [18] point out in their review of impact assessment procedures for CS, there is a lack of standardization for assessing impact. We envision the impact process as a sequence of events: from inputs to a given system (e.g. more OS practices within academia) to immediate outputs (more OA literature, more CS projects), to further impacts beyond the initial system (e.g. increased trust in research or use of research outputs in policy) [16]. The fundamental problem when trying to identify a causal effect of OS on societal impact is that one must compare two situations and study how impacts change: one where the increase in OS practices takes place, and one where it does not. Unfortunately, only in rare circumstances is it possible to observe both situations (e.g. in carefully controlled experiments). Methods to estimate causal effects absent of controlled experiments exist, but require careful reasoning and sometimes strong assumptions. For this reason, we sometimes need to acknowledge that a causal effect cannot be identified, and restrain ourselves from drawing too strong conclusions for policy or advice.

While lots of work has been done by institutions, funders and even publishers to measure progress in the uptake or implementation of OS, much less exists to systematically monitor its societal impact, likely due in part to the difficult nature of establishing causality. In a review focused on broad impacts of OA (academic, economic and societal) by Tennant *et al*. [19], the discussion of societal impact is largely speculative in nature, centring the argument that OA results in increased public engagement with research outputs, absent any evidence to support this claim. Much research exists based on altmetrics, which are designed to measure the presence of published research outside of academia and are taken as an indicator of societal impact [20–22]. Some studies measure the difference in altmetrics (a composite of citations in policy documents, mentions in news or blog posts, social media attention, references in Wikipedia, and readership) between closed and OA publications to indicate the societal impact of OA specifically. We consider these later in this paper. However, altmetrics are questioned as indicators of societal impact for various aspects, e.g. for not giving information on who is engaging or limited coverage [23]. There are some studies that investigate the societal impact of CS broadly, like a survey conducted by Von Goenner *et al*. [24] that found evidence for societal impact stemming from participation, including knowledge and skills acquisition, increased self-efficacy, and a greater interest in science, and some reviews that document the societal impact of CS in focused ways. For example, Aristeidou and Herodotou reviewed research focused on, and documented, changes in learning, awareness and science literacy stemming from online CS [25]. Bonney *et al*. [26] conducted a review of literature focused on similar impacts specific to their connection to data collection and processing within CS projects and presented similar results. Walker *et al*.'s [27] review documented a wide range of both positive and negative impacts derived specifically from CS conducted within water science, including democratization of science, benefits to resource monitoring and management, increased awareness and scientific literacy, increases in social, political and human capital, as well as negative impacts to livelihood and health and safety, among others. Seeking to foster standardization of how the impact of CS is studied, Wehn *et al*. [18] propose categorizing it as impact in society, economy, environment, science and technology, and/or governance (we interpret society, environment, and governance as aspects of societal impact) and offer a wide range of indicators that can be used to measure CS-driven impact. Yet, we note that the evidence of societal impact and methods for measuring it discussed here are limited to CS, and do not necessarily indicate ways of documenting and measuring societal impact of OS as a whole.

Responding to this gap in the literature, we follow the PRISMA Extension for Scoping Reviews methodology (PRISMA-Scr) [28] to systematically scope, critically appraise, consolidate and valorize evidence from the existing literature that demonstrates societal impact of OS generally and its various aspects, including OA, Open and FAIR/Data, Open Methods, Open Code/Software, Open Evaluation and CS. We pose the primary research question (RQ1): *What evidence exists in the literature regarding the effect of OS on the societal impact of research?* In addition, we pose the following secondary research questions:

—
SRQ1: What types of positive or negative, direct or indirect societal impact are observed?
—
SRQ2: What kinds of mechanisms produce them?
—
SRQ3: What specific enabling and/or inhibiting factors (drivers and barriers) are associated with these impacts?
—
SRQ4: What knowledge gaps emerge from this analysis?

## 2. Methods

Following identification of the above research questions, the study proceeded in four steps: identify relevant studies, select eligible studies, extract data from relevant studies and summarize and report the results. The study protocol was pre-registered on 31 October 2022 [29] and an addendum detailing the grey literature and snowball search procedures was published on 29 June 2023 [30], both of which provide deeper methodological detail and are published on the Open Science Framework (OSF) platform. For any changes to what was set out in these documents, see electronic supplementary material, S1.

### 2.1. Identifying relevant studies

We sought to identify all studies presenting evidence (positive or negative) regarding the direct or indirect societal impacts of OS, across all categories of OS practices and types of impact. A search was first conducted for published peer-reviewed literature in the general cross-disciplinary databases Web of Science (WoS) (all databases) and Scopus published between January 2000 and 8 November 2022 (the date of both searches). Search strings were constructed iteratively via keyword/synonym identification and pilot testing. Terms used for types of impact were refined by the team through prioritizing keywords for impact labels applicable generally (engag*, educat*, trust, etc.). We next assessed, given our resources, the maximum manageable number of titles we could screen, and then added new issue-specific keywords (health*, environment*/climat*, covid*/coronavirus) one by one, trialling the search to determine if our maximum number of titles was reached as we went. Since these latter keywords were intended as a supplement to the more general impact keywords, our aim was not to be exhaustive (e.g. including farming or emergencies, for example, would still leave out other domains where science has societal impact and hence potentially OS impact). The COVID-19 pandemic was perhaps the major emergency of our time when we conceived this study, with much discourse on the impact of OS in responding to it. We hence decided to prioritize this as a potential area of societal impact.

The keywords ultimately used to compose the following search strings, beyond OS terms, include:

—
Societal impact
—
Trust
—
Education/understanding
—
Engagement
—
Government policy
—
Sustainable Development Goals
—
Environment/climate
—
Health
—
COVID
—
Participation

Search in both databases took place on 8 November 2022 using the following query details:

**Table:** 

Web of Science (All Databases)—6478 results
(TI = (open scien*’ OR ‘science 2.0’ OR ‘open data’ OR ‘FAIR data’ OR ‘open access’ OR (‘open code’ OR ‘open software’ OR ‘open tool*) OR ‘open method*’ OR ‘citizen science’ OR ‘open peer review’ OR ‘open metric*’) OR AB = (open scien*’ OR ‘science 2.0’ OR ‘open data’ OR ‘FAIR data’ OR (‘open code’ OR ‘open software’ OR ‘open tool*) OR ‘open method*’ OR ‘citizen science’ OR ‘open peer review’ OR ‘open metric*’ OR ‘open access publ*’ OR ‘open access paper*’ OR ‘open access journal*’ OR ‘open access book*’)) AND TS =((impact* OR effect* OR outcome*) AND (engag* OR educat* OR trust OR polic* OR (sdg OR ‘sustainable development goal*’) OR (gender* OR diversit*) OR participat* OR health* OR (environment* OR climat*) OR (covid* OR coronavirus*)))
Timespan: 2000–2022. Databases: WOS, BCI, BIOSIS, CCC, DIIDW, KJD, MEDLINE, RSCI, SCIELO
Search language = English
Scopus—6793 results
TITLE-ABS (‘open scien*’ OR ‘science 2.0’ OR ‘open data’ OR ‘FAIR data’ OR (‘open access’ W/1 publ* OR paper* OR journal* OR book*) OR (open code’ OR ‘open software’ OR ‘open tool*) OR ‘open method*’ OR ‘citizen science’ OR ‘open peer review’ OR ‘open metric*’) OR TITLE (open access) AND TITLE-ABS-KEY ((impact* OR effect* OR outcome*) AND (engag* OR educat* OR trust OR polic* OR (sdg OR ‘sustainable development goal*’) OR (gender* OR diversit*) OR participat* OR health* OR (environment* OR climat*) OR (covid* OR coronavirus*))) AND (PUBYEAR > 1999) AND (LIMIT-TO (LANGUAGE,"English’))

In the second phase of this study, we used ‘snowball search’ to analyse citations to and from included studies, as well as a systematic grey literature search of websites of relevant OS stakeholders (e.g. EC, OECD, UNESCO, etc.), to identify a further 1742 potentially relevant studies. Detailed documentation for both searches, code for the snowball search, and data are included in the data package shared with this paper [31].

### 2.2. Selection of eligible studies

The searches of WoS and Scopus yielded 13 271 total results.

**Figure 1.**
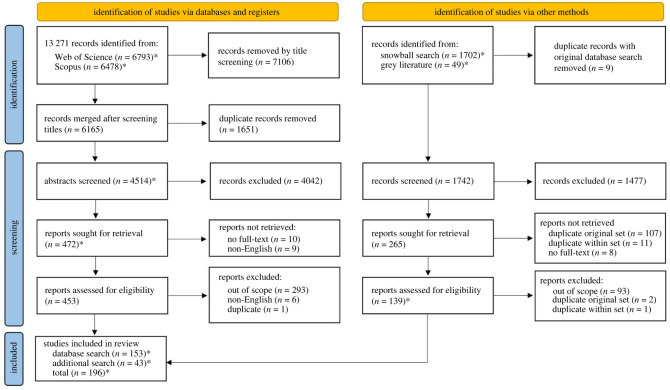
PRISMA-P flow diagram. *Indicates that there is a dataset available at http://doi.org/10.5281/zenodo.10559446 [31] for this step of the process (grey literature records are only available after deduplication with *n* = 40).

Title and abstract screening were guided by the PRISMA-ScR checklist (see electronic supplementary material, S2) and mapped using the PRISMA-P chart (figure 1). The following inclusion criteria were used:

—
Articles on the societal impact of OS (including OA^2^, Open/FAIR Data^3^, Open Methods, Open Code/Software, CS, Open Evaluation).
—
Conducted internationally or nationally.
—
Published from 1 January 2000 until the date of search.
—
Text in English.
—
Full-text available.
—
Study is either a research article, review article, conference paper, or other peer-reviewed output, or a grey literature study from a recognized stakeholder.
—
Study reports evidence of OS societal impact.
—
All methodologies (quantitative, qualitative, mixed, etc.) are eligible.

These criteria were applied in both title/abstract and full-text screening phases. Following an initial screening pass of titles to remove obvious false positives, followed by merging and de-duplication, 4514 records remained from the original search of peer-reviewed literature. Two researchers then conducted title/abstract screening, with the first researcher coding either ‘yes’, ‘no’ or ‘unsure’ for inclusion, and the second researcher then reviewing all entries judged ‘unsure’ to decide inclusion. At this stage, we also recorded the aspects of Open Science (OA, OFD, etc.) to which the included studies were most relevant.

After this, 453 of 472 studies sought were retrieved for full-text screening. All reasonable efforts were made to obtain full-texts, including inter-library loan and emailing authors. Full-texts were imported into a shared Zotero folder. Following full-text screening by one researcher, 153 total studies remained for inclusion from the initial search.

In the snowballing and grey literature phase, 265 studies remained after title/abstract screening, and 43 after full-text screening. Hence, a total of 196 relevant studies were identified for inclusion in this scoping review.

### 2.3. Extracting the data

Data extraction for studies included from the initial search was conducted using a collaborative Excel file shared via Microsoft Teams and carried out according to the data extraction form illustrated in table 1. The studies were assigned to individual co-authors for extraction based on the provisional assignment to which aspect of OS they were primarily relevant. Intermittent checks on data extraction quality were performed by the lead author and feedback discussed within the team. Later, screening and data extraction for the snowballed and grey literature were conducted using the same inclusion criteria and extraction form, but carried out in the Systematic Review Facility (SyRF) online platform [32].^5^

**Table 1.** Categories extracted from included studies in the data charting process.

heading	description
author	name of author/s
date	date article sourced
title of study	title of the article or study
publication year	year that the article was published
publication type	journal, website, conference, etc.
DOI/URL	unique identifier
exclusion	out of scope, non-English, duplicate
justification	if a study was deemed to be out of scope, a justification had to be provided
study details and design (if applicable)	type of study, empirical or review, etc. Notes on methods used in study (whether qualitative or quantitative, which population demographics studied, etc.)
types of data sources included	detail the data sources
study aims	overview of the main objectives of the study
relevance to which aspect of Open Science	Open Access, Open/FAIR Data, Open Methods, Citizen Science, Open Evaluation, Open Science General
relevance to which aspect(s) of societal impact	engagement, participation, education, trust, policy, sustainable development goals, gender, diversity, health, climate/environment, COVID-19^4^
key findings	noteworthy results of the study that contribute to the scoping review question(s)
coverage	optional field to note any relevant information about the level of coverage of the study, e.g. only specific countries, disciplines, demographics covered
confidence assessment	Optional field to note any concerns about reliability/generalizability of findings (e.g. conflict of interest, potential biases, small sample sizes, or other methodological issues) within the study

### 2.4. Summarizing and reporting the results

Data extraction results were collated within two Excel files shared on the Microsoft Teams platform and categorized by aspect(s) of OS (one for the initial database search, one for grey literature and snowballed sources). Co-authors were then assigned to summarize and report results narratively in a shared Google document. For aspects of OS that had many papers, co-authors also summarized the data by societal impact aspect. The team then collaborated to refine this initial narrative and to present it in the form of this paper.

## 3. Results

### 3.1. Overview

**Figure 2.**
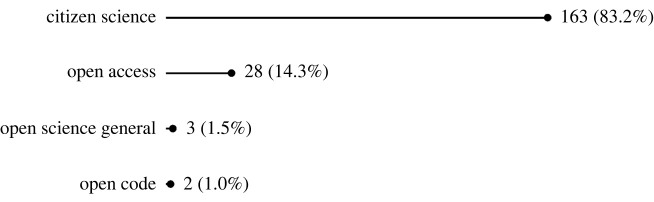
Number of papers by type of OS (% of all papers).

**Figure 3.**
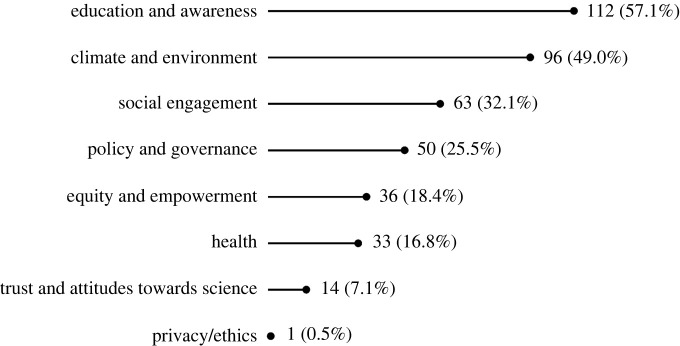
Number of papers by type of impact (% of all papers).

We found 196 papers to be in scope (153 from the original academic literature search, and 43 from grey literature and the academic snowball search). Of these, the vast majority provided evidence of the societal impact of CS (163 papers, 83.2% of OS type instances; figure 2), across a wide variety of types of societal impact (figure 3). Twenty-eight papers demonstrated the societal impact of OA, with impacts including public engagement with scientific literature, use in policy-making, and health-related outcomes. Beyond OA, our search revealed limited evidence of the societal impact of OS. We identified three papers that speak to the impacts of OS in general and two that demonstrate the public health impacts of Open Code/Software. We found no literature with evidence of societal impact from Open Methods, Open Evaluation, or Open/FAIR Data, despite several papers suggesting to do so (see the discussion section).

Education and awareness (112 or 57.1% of papers) and climate and environment (96 or 49.0% of papers) were by far the most commonly evidenced types of impact within our data. Other common types of impact evidenced by our study include social engagement (between citizens and scientists/other stakeholders, with scientific/project outcomes, and with the broader community) (63 or 32.1% of papers), and policy and governance (50 or 25.5% of papers). Less common but also present in the literature are evidence of impacts in terms of equity and empowerment (36 or 18.4% of papers), health (33 or 16.8% of papers), trust in and attitudes toward research (14 or 7.1% of papers), and privacy/ethics (1 or 0.5% of papers).

Looking at the trends within OS aspects (table 2), we found that the majority of papers within CS demonstrate impact in terms of education and awareness (112 or 68.7% of papers), and climate and environment (96 or 58.9% of papers). Frequently, these impacts overlap, with studies demonstrating impacts in education and awareness that pertain to climate and environmental topics. The literature shows that CS also creates impact through fostering social engagement (40 or 24.5% of papers), in the realms of policy and governance (45 or 27.6% of papers) and health (29 or 17.8% of papers), fostering equity and empowerment (36 or 22.1% of papers), and by creating trust in research and impacting attitudes toward it (12 or 7.4% of papers). We found no literature with rigorous evidence of societal impact in terms of diversity or gender.

**Table 2.** Type of impact by OS type, with number of papers coded per intersection (*N*) and % of papers within the OS type category.

OS type	climate and environment	education and awareness	equity and empowerment	health	policy and governance	privacy/ethics	social engagement	trust and attitudes towards research
Citizen Science	58.9% (96)	68.7% (112)	22.1% (36)	17.8% (29)	27.6% (45)	0.0% (0)	24.5% (40)	7.4% (12)
Open Access	0.0% (0)	0.0% (0)	0.0% (0)	7.1% (2)	17.9% (5)	3.6% (1)	78.6% (22)	0.0% (0)
Open Code	0.0% (0)	0.0% (0)	0.0% (0)	100.0% (2)	0.0% (0)	0.0% (0)	0.0% (0)	0.0% (0)
Open Science General	0.0% (0)	0.0% (0)	0.0% (0)	0.0% (0)	0.0% (0)	0.0% (0)	33.3% (1)	66.7% (2)

Papers that demonstrate the societal impact of OA publishing primarily show this in terms of engagement (with OA texts) (22 or 78.6% of papers), but also through policy and governance (5 or 17.9% of papers), and health (2 or 7.1% of papers). One paper provides evidence in terms of privacy/ethics and we found no papers with evidence of OA impact on climate and environment, or education and awareness.

In what follows, we present detailed findings within the OS aspects of CS, OA, Open Code/Software and OS General.

### 3.2. Societal impact of citizen science

#### 3.2.1. Education and awareness

**Figure 4.**
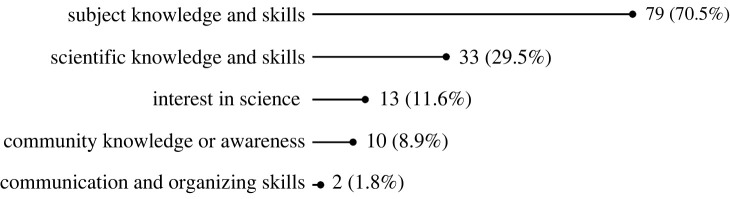
Number and percentage of studies by type of educational impact.

As shown in table 2, the greatest number of papers within CS provide evidence of impact in terms of education and awareness. These impacts were studied across a range of CS projects and programmes, from those in educational settings (from primary school through university) to crowd-sourcing, to community-based initiatives, and across the globe. Most studies in this category used a pre- and post-test methodology (typically surveys, but sometimes also interviews) to evaluate changes to participants' level of subject knowledge, understanding of science and the scientific process, scientific thinking, and/or scientific skills (figure 4).^6^

The majority of these papers demonstrate societal impact by documenting **changes in CS participants’ subject knowledge and skills** associated with the topic of the programme or project in question (79 papers; table 3). Others demonstrated **changes in participants' general scientific knowledge and skills** (33 papers), **changes in participants’ interest in studying science or pursuing a scientific career** (13 papers)^7^, **changes in community knowledge and/or awareness** where the project or programme was situated (10 papers), and changes in **communication and organizing skills** (2 papers). Nearly all of this evidence **indicates positive changes** (table 3), **though a rare few show no impact or mixed results**. Shinbrot *et al*. [107] found a limited impact on knowledge development in an environmental CS programme in Mexico. While Vitone *et al*. [108] established a positive relationship between participation and interest in science and the project subject matter, they found no correlation between participation and subject matter learning. Raddick *et al*. [106] and Meschini *et al*. [81] found no learning gain when CS participation was brief and limited in the nature of participation (in an online galaxy classification project and a tourism-based CS programme, respectively). Though Meschini *et al*. [81] found higher levels of environmental awareness 3 years after participating in a tourism-based CS programme, they did not find evidence of specific knowledge retention. And, while Jordan *et al*. [64] found increases in subject learning, they did not find a gain in *scientific* knowledge. Further, both Derrien *et al*. [49] and Stewart *et al*. [126] found no impact of participation on interest in pursuing a scientific career.

**Table 3.** Literature with evidence of the impact of CS on education and awareness.

	positive impact	no impact	negative impact	total studies
changes in subject knowledge and/or related skills	Adamou *et al.* [33]; Aivelo and Houvelin [34]; Allen [35]; Araujo *et al.* [36]; Aristeidou and Herodotou [25]; Asingizwe *et al.* [37]; Ballard *et al.* [38]; Ballard *et al.* [39]; Branchini *et al.* [40]; Bremer *et al.* [41]; Brossard *et al.* [42]; Carson *et al.* [43]; Chase and Levine [44]; Christoffel [45]; Cronje *et al.* [46]; Damman *et al.* [47]; Dem *et al.* [48]; Derrien [49]; Diprose *et al.* [50]; Ekman [51]; English *et al.* [52]; Forrester *et al.* [53]; Greving *et al.* [54]; Groulx *et al.* [55]; Hadjichambi *et al.* [56]; Haywood *et al.* [57]; Hiller and Kitsantas [58]; Hollow *et al.* [59]; Hoover [60]; Hsu *et al.* [61]; Isley *et al.* [62]; Johnson *et al.* [63]; Jordan *et al.* [64]; Kelly *et al.* [65]; Kermish-Allen *et al.* [66]; Kerr [67]; Kleitou *et al.* [68]; Kloetzer *et al.* [69]; Kobori *et al.* [70]; Lakomy *et al.* [71]; Land-Zandstra *et al.* [72]; Locritani *et al.* [73]; Luesse *et al.* [74]; Lynch-O'Brien *et al.* [75]; Mady *et al.* [76]; Marchante and Marchante [77]; Marks *et al.* [78]; Meixner *et al.* [79]; Merenlender *et al.* [80]; Meschini *et al.* [81]; Nursey-Bray *et al.* [82]; Peter *et al.* [83]; Peter *et al.* [84]; Peter *et al.* [85]; Peters *et al.* [86]; Phillips *et al.* [87]; Popa *et al.* [88]; Santori *et al.* [89]; Schaefer *et al.* [90]; Schlaeppy *et al.* [91]; Schneiderhan-Opel and Bogner [92]; Schuttler *et al.* [93]; Seamans [94]; Seifert *et al.* [95]; Shaw [96]; Silva *et al.* [97]; Stepenuck and Green [98]; Turrini *et al.* [99]; Van Haeften *et al.* [100]; Varaden *et al.* [101]; Von Gönner *et al.* [24]; Walker *et al.* [102]; Walker *et al.* [27]; Williams *et al.* [103]; Zarybnicka *et al.* [104]; Zhang *et al.* [105]	Meschini *et al.* [81]; Raddick *et al.* [106]; Shinbrot *et al.* [107]; Vitone *et al.* [108]	none	79
change in general scientific knowledge and skills	Anderson *et al.* [109]; Aivelo and Huovelin [34]; Ballard *et al.* [38]; Ballard *et al.* [39]; Bedessem *et al.* [110]; Carson *et al.* [43]; Cho *et al.* [111]; Christoffel [45]; Conrad and Hilchey [112]; Cronje *et al.* [46]; da Silva and Heaton [113]; Dickinson *et al.* [114]; English *et al.* [52]; Grossberndt *et al.* [115]; Haywood *et al.* [57]; Hiller and Kitsantas [116]; Hoekstra *et al.* [117]; Isley *et al.* [62]; Johnson *et al.* [63]; Kloetzer *et al.* [69]; Lewis and Carson [118]; Luesse *et al.* [74]; Mady *et al.* [76]; Merenlender *et al.* [80]; Peter *et al.* [85]; Phillips *et al.* [87]; Price and Lee [119]; Ross-Hellauer *et al.* [120]; Trumbull *et al.* [121]; Walker *et al.* [102]; Walker *et al.* [27]; Zarybnicka *et al.* [104]	Jordan *et al.* [64]	none	33
change in interest in studying science or pursuing career in science	Ballard *et al.* [39]; Cho *et al.* [111]; Hiller and Kitsantas [58]; Johnson *et al.* [63]; Koomen *et al.* [122]; Luesse *et al.* [74]; Mahajan *et al.* [123]; Rosas *et al.* [124]; Seifert *et al.* [95]; Vitone *et al.* [108]; Wallace and Bodzin [125]	Derrien *et al.* [49]; Stewart *et al.* [126]	none	13
change in community knowledge or awareness	Asingizwe *et al.* [37]; Ballard *et al.* [39]; Costa *et al.* [127]; Frigerio *et al.* [128]; Johnson *et al.* [63]; Mahajan *et al.* [129]; Schaefer *et al.* [90]; Shinbrot *et al.* [107]; Stepenuck and Green [98]; Walker *et al*. [27]	none	none	10
change in communication and organizing skills	Bonney *et al.* [26]; Kloetzer *et al.* [69]	none	none	2

The findings of some studies indicate elements of CS initiatives that led to positive impacts. Mady *et al*. [76] found that higher degrees of participation in an ornithological CS programme led to greater increases in knowledge, and that these were highest when participants were involved in data collection. Similarly, both Phillips [87] and Ballard *et al*. [39] found that hands-on experience in the research process and interaction with project materials fostered learning, while sustained, long-term participation was found by Bedessem *et al*. [110] to result in increased scientific skills and by Kloetzer *et al*. [69] to positive subject learning outcomes. Dickinson *et al*. [114] observed that community-based projects, specifically, led to gains in scientific capacity, while Von Gönner *et al*. [24] found ‘different forms of social learning, such as systematic feedback or personal mentoring’ to be essential to producing learning gains. Additionally, Frigerio *et al*. [128] found a ‘multiplying effect’ of knowledge gain, wherein knowledge gains by child participants were ‘multiplied’ within the broader community (with definitive post-test results focused on the specific knowledge topic area of the project).

#### 3.2.2. Climate and environment

**Figure 5.**
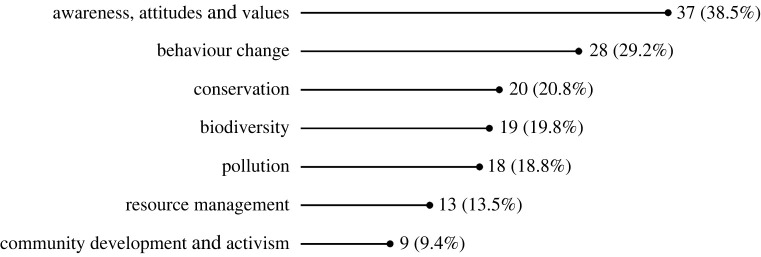
Number and percentage of studies by type of environmental impact.

We identified 96 studies that demonstrate the societal impact of CS on climate and environment. The greatest proportion demonstrates positive impacts on awareness of, attitudes toward and values related to climate and environmental issues (37 papers; approximately 38.5% of this subset of papers). Less than a third (28 papers) demonstrate changes to behaviour as a result of CS activities, and a small selection (9 papers) demonstrate community development around related issues and/or activism stemming from CS activities. Additionally, some studies demonstrate positive impacts on conservation (20 papers), biodiversity (19 papers), counter-measures to pollution (18 papers) and resource management (13 papers) (figure 5).

Studies that demonstrate positive impacts in terms of **changes to awareness, attitudes and values** have shown increases in awareness of, for example, human behavioural impacts on the environment and climate [57,130], development of environmental stewardship values and attitudes [131], changes in attitudes toward particular resources or the environment in general [44], and the development of a ‘green identity’ [107]. In cases where a lesser degree of change or no change was found, authors report that this was a result of participants having pre-existing high levels of awareness and/or already holding strong pro-environmental attitudes [44,53].

Studies that demonstrate **pro-environmental behaviour change** include evidence like changes to personal behaviour that support biodiversity, like gardening in ways that support rather than harm biodiversity [132], changes to farming practices that support ‘climate resilience’ [133], a greater engagement in conservation efforts [134], noticing biodiversity issues and communicating with others about them [64], and changes in decision-making regarding things like waste management, personal consumption, and participating in environmental clean-up efforts [44]. Locritani *et al*. [73] established a causal relationship between increases in knowledge and awareness that result from participation in a CS project or programme and the intention to change one's behaviour. According to a review by Popa *et al*. [88], there is some evidence that certain traits among CS participants predispose them to change their behaviour as a result of participation: namely, these are pre-existing ‘strong environmental attitudes', and involvement in other conservation or research efforts. They also provide evidence that most behaviour changes are private or personal, rather than public-facing, and can be classed as lifestyle changes [88], which suggests that such impact might be limited in scope. Expressing a similar sentiment, Jordan *et al*. [64] classified the behaviour changes they documented as ‘passive’ rather than active (noticing and talking about invasive plants but nothing further). And, in some studies reviewed by Popa *et al*. [88], ‘no significant changes' were documented in terms of pro-environmental behaviour.

Evidence for **impacts to conservation, biodiversity, pollution and resource management** have some overlap with the two areas of impact discussed above, as there is evidence of positive impacts to awareness, attitude, values [48,61,89,104] (for example) and behaviour [89,135,136] (for example) that are relevant to these areas. However, more concrete positive impacts are documented within this evidence, including the use of CS data to inform management decisions and actions [52,65,98,124,129,137–139] (for example), to drive the creation of protected areas and the monitoring of them (and protected species) [68,138,140–143] (for example), the monitoring of and response to pollution incidents [123,144–146] (for example), and to inform relevant policies [129,142,145,147] (for example) though evidence of impact on policies is limited [129]. Further, there is not just evidence that CS *data* serves to create these impacts, but that projects and programmes themselves create community-based monitoring capacity that fills gaps in government monitoring [112,138, 140,143,144,146] (for example).

Providing evidence of the limited impact of CS data, Conrad & Hilchey [112] reported in a review published in 2011 that ‘Many groups find their data is not used in the decision-making process (or published in scientific peer-reviewed journals), either due to data collection concerns or difficulty getting their data to the appropriate decision-maker or journal’. They added, ‘Although there is much anecdotal discussion and website documentation of the environmental benefits of citizen science, more peer-reviewed studies must actually show a relationship between CBM [community-based monitoring] group efforts and environmental improvements to substantiate these claims’. The evidence reported above and included in table 4, published after these findings, suggests that the tide has turned regarding the environmental impacts of CS data (and projects/programmes).

**Table 4.** Literature with evidence of the impact of CS on climate and environment.

	positive impact	no impact	negative impact	total studies
awareness, attitudes and values	Adamou *et al.* [33]; Ballard *et al.* [39]; Branchini *et al.* [40]; Bremer *et al.* [41]; Carson *et al.* [43]; Chase and Levine [44]; Cronje *et al.* [46]; English *et al.* [52]; Evans *et al.* [148]; Fernandez-Gimenez *et al.* [149]; Grossberndt *et al.* [115]; Groulx *et al.* [55]; Haywood *et al.* [57]; Hsu *et al.* [61]; Johnson *et al.* [63]; Kelemen-Finan *et al.* [150]; Kerr [67]; Kleitou *et al.* [68]; Kloetzer *et al.* [69]; Lynch-O'Brien *et al.* [75]; Mahajan *et al.* [123]; Mahajan *et al.* [129]; Marchante and Marchante [77]; Meschini *et al.* [81]; Ostermann-Miyashita *et al.* [131]; Popa *et al.* [88]; Sandhaus *et al.* [151]; Schneiderhan-Opel and Bogner [92]; Schuttler *et al.* [93]; Shinbrot *et al.* [107]; Stepenuck and Green [98]; Toomey and Domroese [152]; Torres *et al.* [153]; Walker *et al.* [102]; Walker *et al.* [27]; West *et al.* [154]	Forrester *et al.* [53]	none	37
behaviour change	Adamou *et al.* [33]; Day *et al.* [135]; Deguines *et al.* [132]; Evans *et al.* [148]; Fulton *et al.* [140]; Gotor *et al.* [133]; Grossberndt *et al.* [115]; Groulx *et al.* [55]; Hadjichambi *et al.* [56]; Haywood *et al.* [57]; Hodgkinson *et al.* [155]; Lewandowski and Oberhauser [134]; Lynch-O'Brien *et al.* [75]; Mahajan *et al.* [123]; Mahajan *et al.* [129]; Marchante and Marchante [77]; Peter *et al.* [83]; Popa *et al.* [88]; Rodriguez *et al.* [136]; Sandhaus *et al.* [151]; Santori [89]; Spellman *et al.* [156]; Stepenuck and Green [98]; Toomey and Domroese [152]; Vasiliades *et al.* [157]; Walker *et al.* [102]; Walker *et al.* [27]	Jordan *et al.* [64]; Popa *et al.* [88]	none	28
conservation	Aceves-Bueno *et al.* [137]; Ballard *et al.* [38]; Ballard *et al.* [39]; Chiaravalloti *et al.* [138]; Crow *et al.* [158]; Day *et al.* [135]; Earp and Liconti [145]; Fulton *et al.* [140]; Haywood *et al.* [57]; Hsu *et al.* [61]; Hyder *et al.* [147]; Johnson *et al.* [63]; Mwango'mbe *et al.* [141]; Pecorelli *et al.* [159]; Sandhaus *et al.* [151]; Santori *et al.* [89]; Schlaeppy *et al.* [91]; Skrbinsek *et al.* [160]; Soroye *et al.* [142]; Zhang *et al.* [105]	none	none	20
biodiversity	Branchini *et al.* [40]; Carson *et al.* [43]; Deguines *et al.* [132]; Dem *et al.* [48]; Earp and Liconti [145]; Fraisl *et al.* [161]; Hyder *et al.* [147]; Jordan *et al.* [64]; Kelemen-Finan *et al.* [150]; Kleitou *et al.* [68]; Lee *et al.* [162]; Lynch-O'Brien *et al.* [75]; Marchante and Marchante [77]; Peter *et al.* [84]; Peter *et al.* [85]; Schlaeppy *et al.* [91]; Shaw [96]; Soroye *et al.* [142]; Zarybnicka *et al.* [104]	none	none	19
pollution	Ballard *et al.* [38]; Brooks *et al.* [144]; Dhillon [163]; Earp and Liconti [145]; English *et al.* [52]; Gray *et al.* [146]; Grossberndt *et al.* [115]; Hodgkinson *et al.* [155]; Hyder *et al.* [147]; Land-Zandstra *et al.* [72]; Mahajan *et al.* [123]; Mahajan *et al.* [129]; Popa *et al.* [88]; Rodriguez *et al.* [136]; Ruppen and Brugger [164]; Segev *et al.* [143]; West *et al.* [154]; Zettler [165]	none	none	18
resource management	Ballard *et al.* [39]; English *et al.* [52]; Fernandez-Gimenez *et al.* [149]; Hyder *et al.* [147]; Kelly *et al.* [65]; Kobori *et al.* [70]; Meixner *et al.* [79]; Merenlender *et al.* [80]; Njue *et al.* [139]; Shinbrot *et al.* [107]; Stepenuck and Green [98]; Walker and Smigaj *et al.* [27]	Conrad and Hilchey [112]	none	13
community development and activism	Dhillon [163]; Fernandez-Gimenez *et al.* [149]; Rosas *et al.* [124]; Sandhaus *et al.* [151]; Popa *et al.* [88]; Ruppen and Brugger [164]; Shinbrot *et al.* [107]; West *et al.* [154]; Zhang *et al.* [105]	none	none	9

A minority of studies (nine) provide evidence that CS can result in **community development** [105,149,151,163] and/or **activism** around the issue in focus [88,107,124,154,163,164].

#### 3.2.3. Policy and governance

Of the 45 papers that demonstrate policy and governance impacts (27.6% CS papers), the majority relate to impacts realized at local and regional levels, with fewer related to national or international policy and governance arenas. Most of these papers reported on projects within the domain of climate/environment (33 papers) (table 5), with a few others focused on health [35,52,78,124,143,173,175,177] and infrastructure [124,173,175]. We found evidence for a range of impacts, but the majority reported on the use of CS data by government agencies to monitor or manage natural resources, environmental and health risks, and the built environment (32 papers). Of these, two papers provided evidence that CS data are in use in monitoring Sustainable Development Goal (SDG) indicators specifically [142,161]. In addition, some found evidence of CS leading to the development of new management techniques [94,96]. Yet, there are challenges to integrating CS data into monitoring and management due to a lack of ‘official recognition’ of it [140] and a ‘lack of systems in place within […] agencies for integrating community data into environmental reporting’ [86].

**Table 5.** Literature with evidence of the impact of CS on policy and governance.

	positive impact	no impact	negative impact	total studies
climate/environment	Aceves-Bueno *et al.* [137]; Ballard *et al.* [38]; Barnard *et al.* [166]; Bremer *et al.* [41]; Brooks *et al.* [144]; Chandler *et al.* [167]; Chiaravalloti *et al.* [138]; Couvet *et al.* [168]; Crow and de Groot [158]; Dhillon [163]; English *et al.* [52]; Friedman and Rosen [169]; Fulton *et al.* [140]; Hollow *et al.* [59]; Hyder *et al.* [147]; Ierodiaconou *et al.* [170]; Kobori *et al.* [70]; Mahajan *et al.* [129]; Mwango'mbe *et al.* [141]; Newman *et al.* [171]; Njue *et al.* [139]; Pecorelli *et al.* [159]; Peters *et al.* [86]; Ruppen and Brugger [164]; Seamans [94]; Segev *et al.* [143]; Shaw [96]; Skrbinsek *et al.* [160]; Soroye *et al.* [142]; Stepencuk and Green [98]; Stepenuck and Genskow [172]; Walker *et al.* [27]; Walker *et al.* [102]; Zettler *et al.* [165]			33
monitoring and management of resources	Aceves-Bueno *et al.* [137]; Allen [35]; Ballard *et al.* [38]; Brooks *et al.* [144]; Chandler *et al.* [167]; Chiaravalloti *et al.* [138]; Couvet *et al.* [168]; Crow and de Greeot [158]; English *et al.* [52]; Fulton *et al.* [140]; Hyder *et al.* [147]; Ierodiaconou *et al.* [170]; Kelly *et al.* [65]; King *et al.* [173]; Kobori *et al.* [70]; Mahajan *et al.* [129]; Mwango'mbe *et al.* [141]; Newman *et al.* [171]; Njue *et al.* [139]; Pecorelli *et al.* [159]; Peters *et al.* [86]; Rosas *et al.* [124]; Ruppen and Brugger [164]; Seamans [94]; Segev *et al.* [143]; Shaw [96]; Skrbinsek *et al.* [160]; Stepencuk and Green [98]; Stepenuck and Genskow [172]; Tuckett *et al.* [174]; Walker *et al.* [27]; Zha *et al.* [175]	Fulton *et al.* [140]; Peters *et al.* [86]		32
policy	Ballard *et al.* [38]; Barnard *et al.* [166]; Bonney *et al.* [26]; Bremer *et al.* [41]; Chandler *et al.* [167]; Dhillon [163]; English *et al.* [52]; Friedman and Rosen [169]; Hollow *et al.* [59]; Hyder *et al.* [147]; Kobori *et al.* [70]; Mahajan *et al.* [129]; Marks *et al.* [78]; McGreavy *et al.* [176]; Mwango'mbe *et al.* [141]; Rubio *et al.* [177]; Soroye *et al.* [142]; Stepenuck and Green [98]; Zettler *et al.* [165]	Fulton *et al.* [140]; Segev *et al.* [143]; Von Goenner *et al.* [24]		22

Fewer papers reported results in terms of policy development (22) and among these, just three documented CS impact in the creation of or changes to legislation [52,165,176]. While 18 papers demonstrated CS projects, participants and/or findings having an impact on policy development, including positive impacts on education and awareness among policy-makers [165], according to reviews conducted by Mahajan *et al*. [129] and Stepenuck & Green [98], evidence of policy impact is limited and reports suggest that it is difficult to achieve. Reporting on a study conducted on water quality in rural Maine, USA, Segev *et al*. [143] found that policy impact may be hindered by political and corporate interests that conflict with CS findings. Von Goenner *et al*. [24] reported that participants believe that their data are under-used in policy-making and Fulton *et al*. [140] found that getting official recognition of CS fisheries data at the national policy level in Mexico can be difficult, though it is impactful at the local level by informing the creation of ‘no take zones’ and setting catch limits.

#### 3.2.4. Social engagement

About a quarter of papers (76) within CS demonstrate impact in terms of social engagement. These include the fostering of engagement between CS participants and other stakeholders either within the research context or as a result of it (14 papers), and project and participant engagement with the broader community (29 papers). To the first point, there is evidence that participation in CS strengthens relationships between project stakeholders [51,67,86,127,154,176–178], increases trust among them [149], allows for the creation of ‘peer-to-peer networks' [114], fosters interpersonal relationships between participants [153] and new relationships and collaborations between stakeholder organizations [100,105].

More evidence demonstrates that CS promotes engagement with the broader community in a variety of impactful ways. There is considerable evidence that participants of CS share their knowledge, project results and practical skills with their families, networks and communities, communicating about the project or programme itself, or its scientific findings [35,53,57,62,63,75,101,128,148,156,162,177]. Studies also show that CS fosters engagement of the broader community *in* the programme or project and its outcomes [57,91,136,151,154,156,177,179]. Evidence also demonstrates that CS fosters and strengthens social ties and community. Participation in CS leads to further community engagement on the part of participants [50,136,154,156,173,179], stronger ties to place and connection with community [57,69,105,148,151,173], and has been shown to increase social capital [27,45,64,112,114,172,180].

There is also evidence that CS can result in the weakening of social ties between participants and the weakening of community. In some cases, engagement may result in conflict (when findings pit the interest of one group against another [27,102] or when resources are at stake [178], for example), the erosion of social capital (e.g. when the local knowledge of participants is not valued by researchers) [27], and the over-burdening of the public with responsibilities that should lie with governments [27,102].

#### 3.2.5. Empowerment and equity

Evidence exists that CS can empower participants and communities and foster equity (36 papers, 22.1% of CS papers). CS data and/or project results can empower participants and community members to advocate for their interests in interaction with decision-makers [35,71,78,102,114,120,163,164, 172,177,181], contribute to decision-making processes [98], monitor the state of their environment [27,140,143,172], and to pursue and implement solutions to problems [156,182].

Evidence also shows that participation in CS can lead to participants developing leadership capacity and taking on leadership positions within projects and their communities [27,52,143,176] and increases in self-efficacy among adult and student participants (a person's belief in their ability to do certain things in order to achieve certain goals) [37,57,58,63,74,76,84,95,107,122,150,151,179,183–185]. Additionally, in a case documented by Hoover [60], project training empowered participants through career development.

In terms of equity, evidence shows that CS can achieve environmental justice in the context of environmental inequality, e.g. by returning rights over traditional fishing territories to communities [138] or improving neighbourhood infrastructures [124,163,173]. Yet, Tubridy *et al*. [186] observed that CS can in some cases ‘compound inequalities by transferring responsibility and blame for air pollution to those who have limited resources to address it’, and Walker *et al*. [27] documented similar evidence in their review paper. Additionally, some studies have documented that CS participant demographics overall point to inequitable participation opportunities, with wealthier and more privileged people more often targeted and better able to participate (in terms of time and resources) [27,120,157].

#### 3.2.6. Health

We identified 29 papers that provide evidence regarding the health impacts of CS (27.8% of CS papers). These papers are related primarily to environmental health risks (air quality, pollution, pests, etc.) but also to physical health (fitness, food and gardening, chronic disease prevention, etc.). The largest number of papers (9) illustrate positive impacts of the CS programme/project itself on the health and safety of participants and their communities [27,124,136,144,155,173,177,181,187]. Evidence also shows that CS participation leads to changes in behaviour that benefit health [60,62,78,129,188], including using project-generated air quality data to determine when to engage in outdoor activity [123] or choosing to cycle or walk rather than drive to improve air quality [155]. Yet, van der Feltz *et al*. [189] found no evidence of impact but cited the low intensity of the programme in question as the likely reason for this. Additional evidence demonstrates that CS effectively spreads awareness of health risks and ways to avoid them [37,62,72,95,123,190] and that it can lead to changes in the lived environment that support improved health and safety [143,164,173,175].

Two papers demonstrated negative health impacts. In a review paper, Walker *et al*. [27] reported evidence that participation in some CS activities poses health and safety risks to participants (e.g. conservation monitoring in remote and/or dangerous locations), while Simmons *et al*. [187] documented a risk of burnout and ‘secondary trauma’ to participants in an online crowd crisis-mapping project that was used to provide real-time emergency response in the aftermath of a disaster.

#### 3.2.7. Trust and attitudes toward research

Several papers (12) demonstrated the impact of CS in terms of trust between scientists and others, and attitudes toward research in general. Bruckermann *et al*. [191], Christoffel [45] and Price & Lee [119] provide evidence that participation in CS leads to more positive attitudes toward research. Other studies have demonstrated that CS establishes trust between researchers and other stakeholders [45,82,112,140,149,178,190,192], and that it increases trust in research [102] and in local knowledge [27]. Yet, as Walker *et al*. [27] point out in their review paper, there is also evidence that trust between researchers and other stakeholders can be damaged through CS when problems amongst stakeholders or with the project outcomes arise.

### 3.3. Societal impact of Open Access

Investigating the impact of OA in terms of **social engagement**, multiple studies demonstrate a general altmetric advantage for OA journal articles by investigating the composite ‘Altmetric Attention Score’ (AAS). In their systematic review, Araujo *et al*. [193] reported on two studies showing a general ‘Open Access Altmetrics Advantage’ (OAAA) [194]. Additionally, Clayson *et al*. [195], Long *et al*. [196] and Yu *et al*. [197] reported an OAAA in their respective fields of research. These studies demonstrate overall greater attention to OA publications on a variety of (non-academic) platforms and outlets (not further differentiated by platform or audience (see Discussion)). Other studies regarded only certain aspects of altmetrics. OA articles have been found to receive more attention on social media (especially on Twitter (now X), but also Facebook) and more mentions in blog posts [198–205]. They are also more present in news outlets [200,205–207], are engaged with more on Mendeley [198,201,203], and are more often referenced on Wikipedia [208] compared to closed-access articles. Similar patterns are observed for books across the same platforms and outlets [209–211].

The breadth of coverage varies greatly with studies investigating the relationship of OA status and altmetrics either globally (e.g. [208]), for a specific field (e.g. [195]), or in comparison between different areas of research. Hadad & Aharony [204], for instance, reported a field-specific advantage for captures, mentions and social media attention for science, technology, engineering and mathematics (STEM), but not social science and humanities (SSH), journal articles. Multiple studies also distinguished between different types of OA. While Long *et al*. [196] found an altmetrics advantage only for gold OA (not green OA), Clayson *et al*. [195] reported a smaller but still significant effect for green OA. Maleki [203] reported increased attention on Mendeley only for preprints, and more Twitter mentions only for gold OA, compared to closed access. The reviewed literature indicates that societal impact investigated through altmetrics might be specific to certain platforms/outlets, disciplines or types of OA.

Three studies in our set investigate topics related to those of altmetrics research, i.e. also focus on outreach of research or engagement of the broader public. In a matched case-control analysis, links to articles on a journal's social media page were clicked more often if they were indicated as OA compared to paid content [212].^8^ When scholarly books are published OA, they generally have a larger and geographically more diverse readership than closed-access books and are accessed more frequently in low- and middle-income countries [213]. However, with both these studies, further demographics of readers remain unclear. Fleerackers *et al*. [214] focused in their literature review specifically on journalists' use of OA publications and preprints. They concluded that journalists rely more on other criteria to evaluate the quality of sources (e.g. impact factor) and are concerned about the trustworthiness of OA publications, therefore only making limited use of them. This appears to be in contrast with findings on OA publications being more present in mass media (see above).

Evidence of **policy impact** stemming from OA is mainly explored in studies framed as altmetrics investigations. Policy impact is thereby measured through citations of OA literature within policy documents. Comparing citations of journal articles by policy documents, Tai & Robinson [205], Vilkins & Grant [215], and Zong *et al*. [216] found an advantage for OA over closed access. This indicates that OA publications were consulted more often as a reference by policymakers. Besançon *et al*. [217] report that some preprints on COVID-19 had already been included in policy documents before being retracted due to quality concerns. This finding demonstrates a potential negative societal impact of preprints, a form of open publishing.

Evidence on the further societal impact of OA publishing is thin and often only anecdotal. Regarding the impact on **health or healthcare**, one randomized experimental study found that mental health professionals gained more knowledge when an article they were asked to read was freely accessible [218]. There were some indications that treatment recommendations within the study were impacted more when access to the resource was free. In their literature review, Davis & Walters [219] reported in 2011 that they found no additional studies on the impact of OA on clinical decision-making and that no study had yet investigated the use of OA biomedical literature by the broader public. One single study found medical images of transgender patients to be openly available on Google Images more often when they were published within an OA article compared to a non-OA article, showing potentially greater negative impact in the area of **privacy and ethics** if appropriate participant consent was not established [220].

### 3.4. Societal impact of other aspects of Open Science

For some aspects of OS, far fewer relevant articles were found. Three articles were identified as relevant to the societal impact of OS in general. One paper, by Zong *et al*. [221], indicated impact in terms of **social engagement**. The authors analysed articles from ‘psychological science’ between 2014 and 2021, finding that OS badges were correlated with increased social media attention. Two papers examined aspects of societal **trust in scholarly work**. Rosman *et al*. [222] examined OS's relationship to public trust in research in two studies. In the first survey study (of participants from a German general population sample), they found that OA and other OS practices are rated by the majority of participants as important and as increasing their trust in the scientists. In a second experimental vignette study, participants were presented with descriptions of research that signalled or did not signal the use of OS practices. Effects on trust were not conclusive across the two conditions, although the authors did interpret some indications of enhanced public trust when OS practices are employed. Similarly, Song *et al*. [223] performed pre-registered experiments examining public perceptions of studies employing OS practices. OS research and researchers were perceived as ‘more credible and trustworthy’ than non-OS counterparts by their cohort of members of the American general public.

Two relevant articles relating to Open Code and Software were identified, demonstrating **health** impact. Bokonda *et al*. [224] performed a (non-systematic) literature review to synthesize findings regarding the adoption of Open Data Kit (ODK), an Open Source suite of tools for data collection and sharing that is free and does not require certification or a stable Internet connection for usage and is hence of particular use in developing countries. They found that this Open Source platform appeared to be most relevant in health contexts, with 11 of the 15 included papers in this area, and the remaining from agriculture (*n* = 2), fisheries (*n* = 1), and the ‘social domain’ (*n* = 1). They concluded that ODK has been used in Kenya, Mali, India, Nigeria, Ethiopia, Madagascar, Tanzania, Mozambique and the Dominican Republic, where it has ‘helped to improve many health programs and systems’. Kobayashi *et al*. [225] performed a narrative review of recent works related to the use of Open Source Software for the COVID-19 pandemic. They found that Open Source projects including GNU Health, OpenMRS, DHIS2 and LIFE took actions enabling various activities (e.g. contact tracing, epidemiological reporting, and laboratory test management, among others).

## 4. Discussion

The primary aim of this scoping review was to identify and synthesize the evidence of the societal impact of OS (RQ1). Our findings show that OS generates societal impact in terms of education and awareness, climate and environment, engagement, policy and governance, equity and empowerment, health, and trust and attitudes toward research. These impacts are primarily direct, yet some indirect impacts were also identified (table 6) (SRQ1). Here, by ‘direct impacts’, we refer to those that are directly created by an OS practice, like how participation in a CS initiative leads to changes in behaviour related to the issue in focus by the initiative. By ‘indirect impacts’, we refer to those that follow on from a direct impact. For example, biodiversity or pollution in a community may be positively impacted by changes to behaviour that directly stem from participating in a CS initiative.

**Table 6.** Direct and indirect impacts evidenced in the literature and reported in the Results section, with OS type indicated.

direct impacts	indirect impacts
**education and awareness**	
increase in subject knowledge and skills (CS) increase in scientific knowledge and skills (CS) increase in interest in science (CS) increase in community-level knowledge and awareness (CS)	increase in knowledge and awareness within social networks and families of participants (CS)
**climate and environment**	
changes to awareness, attitudes and values (CS) changes to behaviour (CS) positive impacts on conservation (CS, direct programme/project action) positive impacts on biodiversity (CS, direct programme/project action) positive impacts on resource management (CS) community development and activism directly resulting from the project/programme (CS)	positive impacts on conservation (CS, indirect impact of changes to behaviour) positive impacts on biodiversity (CS, indirect impact of changes to behaviour) community development and activism indirectly following a project/programme (CS)
**social engagement**	
engagement/relationship building between CS participants and other stakeholders (CS) engagement with the broader community about the project/programme, expert knowledge and results (CS) bringing more community members into the programme/project (CS) strengthens community (CS) increase in social capital (CS) greater societal engagement with research outputs/knowledge (OA, OS general (badges))	*no evidence found*
**policy and governance**	
enabling monitoring and management of natural resources, environment, and health risks, including SDG indicators (CS) creation of new management techniques (CS) creation or changes to legislation (CS) positive impact on knowledge and awareness among policy-makers (CS) increased integration of research in policy-making (OA)	*no evidence found*
**health**	
improvement to health and safety of participants and communities (CS) beneficial behaviour change (CS) increased awareness of health risks and how to mitigate them (CS) increased knowledge among healthcare providers (OA) changes to healthcare treatment guidance (OA) improvement to healthcare delivery and public health management (OCS)	*no evidence found*
**empowerment and equity**	
creation of skills and capacity to monitor environment/issues (CS) development of leadership capacity and skills (CS) increase in self-efficacy (CS) environmental justice through community improvements (CS) more diverse readership (OA)	career development (CS) taking on leadership roles in community (CS) advocate for interests with decision-makers (CS) contribute to decision-making processes (CS) return of land and resource rights (CS)
**trust in and attitudes toward research**	
creation of trust between researchers and other stakeholders (CS) greater trust in research (CS, OS general) more positive attitudes toward research (CS, OS general) greater trust in local/indigenous knowledge (CS)	*no evidence found*

Notably, our findings also show that the evidence presented in table 6 is primarily attributed to the impact of CS, specifically, with some evidence of impact from OA publishing and little evidence for other aspects of OS (table 2). The evidence is also clustered within particular *types* of impact, with most of it showing impact in education and awareness, climate and environment, and engagement (table 2). We emphasize that these findings indicate what is currently evidenced in the literature surveyed here, and that it is likely that more and other societal impacts from OS exist, both direct and indirect. It appears, though, that they have either not yet been studied and/or documented, or that we did not find them with the methods we deployed. Wehn *et al*. [226] offer some insight into why the dearth of evidence of OS impact in changing policy or fostering new policy exists, for example. They point out that the ‘increasing complexity and opacity of policy-making processes’, coupled with the often long time gap between the conclusion of a project and anything that may manifest in policy as a result, make it hard to establish this type of causal impact (driven by CS, in their case). We turn our attention to additional barriers to assessing impact later in this discussion.

We are able to identify particular mechanisms that are responsible for generating some of the impacts identified (SRQ2). For impacts generated by CS, the evidence shows that public participation in research, the collaborative creation of data, the uptake of these data, and stakeholder engagement within such projects and programmes are mechanisms which lead to various types of societal impact. Further, there is evidence that the participation of the public in research leads to every type of impact identified by this review. In terms of CS data, evidence shows that their creation serves unmet data needs in a variety of contexts and that their uptake is impactful in policy, governance, and the empowerment of citizens and communities. Additionally, the mechanism of stakeholder engagement in CS projects is shown to lead to strengthened social ties and communities, equity and empowerment.

There is also evidence that indications of OS practices, like OS badges, lead to greater trust in research. Similarly, the results show that indications of OA for publications lead to greater engagement with research. Yet, we note, as other critics of altmetrics have, that the studies included in this review are unable to provide evidence of *who* is engaging with OA publications. The greater degree of engagement is unquestioned, but whether it is evidence of *societal* impact remains an open question. If readers of OA publications are primarily scientists, then societal impact via ‘public’ engagement with these texts is limited.

Our findings also illuminate some enabling and inhibiting factors that influence the societal impact of OS (SRQ3) (table 7). The evidence pertaining to CS reveals that project or programme characteristics, including the depth and duration of participation [69,76,87,110], the interactions between scientists and participants (and between participants) influence the extent of changes to education and awareness [24]. The environmental impact of CS is influenced by whether or not the project or programme responded to a community need for data [68,123,138,140,141,143–146] and the extent to which policymakers and administrators are willing to accept this data and have mechanisms in place for using them [24,140,143]. Cutting across impact types, a project or programme being driven by community need is an enabling factor for impact [114,124,138,143,163,164,173,175]. Evidence for other OS aspects is more limited, but some findings pertaining to OA suggest that the type of OA (green versus gold) [195,196], the specific social media platform or website (e.g. [198,199,203]), and clear signalling of OA status [212] are factors which influence social engagement with OA outputs (possibly in interaction with research fields).

**Table 7.** Enabling and inhibiting factors for societal impact of OS.

enabling factors	inhibiting factors
**education and awareness**	
CS	CS
duration of participation depth of participant engagement in the research process community-based or driven projects feedback to and mentoring of participants peer-to-peer learning integration of families and social networks	shallow, inconsistent or short-term participation
**climate and environment**	
CS	CS
creation of needed data availability/interest of community/participants responding to community needs pre-existing pro-environmental attitudes among participants policy and administrative acceptance of CS data policy and administrative mechanisms for using CS data	shallow, inconsistent or short-term participation
**social engagement**	
CS	OA
stakeholder management collaborative/power-sharing approach	lack of trust in OA among journalists
OA	
research field (STEM versus SSH) type of OA (gold versus green) social media/Web platform clearly signalling OA status in social media posts country-level economic status	
**policy and governance**	
*no evidence found*	CS
	lack of official recognition of CS data lack of systems in place to integrate CS data political interest corporate interest/lobbying
**health**	
CS	
responding to a problem/community need directly involving community in the project/programme OCS	*no evidence found*
dissemination of open tools	
**empowerment and equity**	
*no evidence found*	*no evidence found*
**trust in and attitudes toward research**	
OS general	
awareness of OS among the general public	*no evidence found*

While our findings demonstrate a wide variety of societal impacts derived from OS practices, they also illuminate considerable knowledge gaps (**SRQ4**). Strikingly, the evidence we gathered is concentrated around CS, and further, mostly focused on impacts derived from *participation in CS*, rather than those derived from the *research generated by CS* (though some evidence of this does exist). Elucidating the challenges of assessing the impact of CS, Wehn *et al*. [18] point out that those leading CS projects may find it challenging to measure medium- and long-term impacts due to the disconnect between the timeline of such work and project funding structures. Assessing impacts necessarily comes after a project has ended, which means the funding linked to the project has ceased to be available. This aligns with the conceptualizations of societal impact offered by the LBG [1] and Bornmann [2], which emphasize the long-term nature of impacts and the process of tracking them. Additionally, Wehn *et al*. [18] found through their review of CS impact assessment studies that project priorities and a lack of assessment competences within the project team are barriers to this kind of work. Further, as mentioned above, our review returned limited evidence of the societal impact of OA and other OS aspects. We imagine that Wehn *et al*.'s [18] observations extend to assessing the societal impact of projects that produce other OS outputs, such as Open Code or Software, as well. From this, we might suggest that once broader frameworks for assessment of OS impact are in place (discussed below), funding instruments may make specific provisions for projects to ensure consistent data collection to facilitate longer-term impact assessment.

Also striking is the sheer absence of evidence of societal impact derived from Open/FAIR Data within the surveyed literature. Throughout this study, we considered 250 texts focused on OFD (after title screening) that represented a diversity of research areas and aims but found that any claims of societal impact were speculative rather than based on observed and documented usage. For example, *possible* impacts included potential privacy violations [227,228], improvements in health research [229–231], or better monitoring of SDGs [232], yet evidence to back these claims was not presented.

It is important to note, as stated in our Methods section, that we excluded Open Government Data (OGD) from our study. Our study focused on OS practices within academic research, and therefore societal impact from OGD was out of scope. We note, however, that there does appear to already be substantial literature focused on the societal and economic impact of this type of open data (we caught much of it in our initial search of the academic literature). Considering the methodologies deployed to study it may prove instructive for new research into the societal impact of Open/FAIR (academic) data. While case studies are often employed due to the complexity of impact assessment (See, e.g. [233,234]), attempts have also been made to quantify the societal impact of OGD, for example, by the Open Data Barometer^9^, which mainly uses expert surveys on topics such as environmental impact or the inclusion of marginalized groups as indicators for societal impact.

Overall, it appears that the evidence included in this study is concentrated in areas where establishing evidence of OS societal impact is less challenging due to established methodologies or datasets. The majority of our evidence is generated through CS projects and programmes and focused on learning impacts because there are established methods for conducting pre- and post-test surveys with participants and communities and these can be done with participants from any CS initiative. Additionally, there is considerable evidence of climate and environmental impact from CS because CS is an established approach to responding to problems that fall within these realms, by, for example, generating needed but missing monitoring data or pushing back on community-level environmental injustices. Similarly, there are established methods and workflows for tracing OA publication references, online engagement with them, and online interactions about them; therefore, numerous studies can harness and make use of altmetrics data (questionable though the veracity of societal impact as measured by this indicator may be).

Much more challenging is tracing the usage and societal impact of OFD and Open Code/Software. A lack of consistent referencing practices for these resources across academic disciplines and research fields makes it extremely challenging to understand usage and impact within academia, and the societal impact that may stem from research that uses these resources. And, while one might be able to classify those who view and download open resources based on IP address or other user details, this would still be several steps away from creating evidence of use and societal impact. In addition, delineating which contributions to Open Source projects come from academia is also difficult since (especially larger OS projects) often are the outcome of contributions from diverse contributors. Many researchers no doubt contribute to Linux, for example, but it was started by someone who did not continue in academia beyond their master's degree [235]. The big tech players also invest heavily in OS development. Even projects which are used intensively within research (e.g. Sci-Kit Learn in Machine Learning^10^) are often developed by non-academics (Sci-Kit Learn was developed at Google Summer of Code by a non-academic [236]). That separating out contributions from the academic research community, as opposed to others, is problematic may also explain why we did not identify any distinct evidence on this within our reviewed literature.

Our study reveals that knowledge gaps also exist around causation. While some of the evidence included here is causal, i.e. there is an established causal relationship between an OS practice and a type of societal impact, the majority of the evidence included in this study is correlational. For example, among all the included studies on the societal impact of OA, only two out of 28 used a research design permitting causal claims, while all others were observational in nature. More research on *causal* relationships between OS interventions, activities, outcomes and impact is therefore needed to meet the institutional and governmental desire to monitor the impact of OS (See Klebel and Traag [237] on how to incorporate causal thinking into empirical studies on science). Yet, establishing causality in this sense is not necessarily about establishing linearity. As Wehn *et al*. [18] point out, establishing causality may involve both ‘intermediary outcomes and impacts within a given domain’ as well as ‘between outcomes in different domains.’ Similarly, Coulson *et al*. [238] documented through research that ‘pathways to change and impact are opened by enabling the step from awareness to action,’ pointing to a step-wise approach fostering societal impact and tracking it in a causal way [239]. Further, Wehn *et al*. [18] caution against creating ‘impact silos’ in assessment work, where a lack of awareness exists between interrelated interventions, outcomes, and impacts that may occur across various domains (e.g. between social changes and environmental impacts).

We emphasize, though, that this work should not only be done on an ad-hoc project basis, for reasons established by Wehn *et al*. [18]. While some projects may have the funding, capacity and time to establish evidenced-based indicators of societal impact, there is need for more broad-based impact monitoring frameworks to do this work. At present, infrastructures for OS monitoring whether national (e.g. French Open Science Monitor^11^) or international (e.g. COKI's Open Access Dashboard^12^) seem to focus primarily on monitoring the uptake of OS practices. Yet, monitoring whether more researchers are publishing more OA, or sharing more research data, while important, does not answer the original aims motivating the implementation of OS – whether scientific publications are used by more diverse audiences (e.g. academic periphery, lay publics, industry); whether OFD is being used to fuel scientific or economic innovation; whether fostering transparency and sharing is raising standards of quality in research. OS monitoring frameworks and infrastructures must, as OS become mainstream, begin to answer such questions. The PathOS project, for which this review was conducted, aims to contribute to this work by establishing evidence-based, causal impact pathways for OS through modelling and case study implementation. We situate the intended contributions of this project alongside those of others who have already demonstrated that it is possible, for example, to link CS outcomes to SDG indicator monitoring [240], for co-created community-level indicators to measure both short and long-term impacts of CS initiatives [238], and that there are evidenced-based principles for assessing CS impact [18] that can reasonably be extended to assessing the societal impact of OS broadly speaking. In anticipating and contributing towards the further development of frameworks and infrastructures, we especially look forward to the work of the UNESCO working group on OS monitoring [241].

Though there is considerable work to be done in this area, we believe that the results of our study fill an important gap in the literature. Though most of the evidence that we found demonstrates short-term rather than long-term impact, our findings validate and expand upon the more focused and subject-specific reviews of societal impacts driven by CS [25–27], and build on Tennant's [19] prior review of OA by demonstrating that evidence of societal impact from OA publishing remains lacking, eight years later. And, though the evidence outside of CS is limited, we also demonstrate that multiple aspects of OS can contribute to the same categories of societal impact.

We note that this study, while intended to be a wide-reaching synthesis of published evidence of societal impact of OS, does have some limitations. Included studies are limited by language and (possibly) publication venue (due to the use of exclusive academic databases for the initial search). The parameters of our search did not overtly include other OS practices, like preprints, preregistration, open analysis, and open collaboration, therefore we may have missed evidence of societal impact stemming from these. In addition, our search string—although covering widely used general terms for types of impact (e.g. trust, education, etc.) – included only some keywords related to distinct domains or issues (e.g. health, climate, COVID) but not others such as farming or emergencies. We acknowledge that these pragmatic choices (made with the aim of keeping the number of included titles within a manageable amount given available resources) might mean that our search strategy failed to capture all impact studies (if they did not use more general terms for impact within their titles or abstracts). We further note that both qualitative research and arts and humanities have low representation within the corpus of literature included in this study, therefore evidence of societal impact stemming from OS within these realms may have been missed. We recognize that publication bias toward positive results is a known problem within scientific research, and therefore expect that we may be missing evidence of null or negative societal impact. And importantly, we acknowledge our authorship team's collective positionality as white Europeans has shaped our research process such that our conceptualization of societal impact and evidence of it may not be as robust and nuanced as it could be.

## 5. Conclusion

In sum, there is considerable evidence within academic and grey literature of the societal impact of OS, but it is almost entirely derived from studies focused on the impact of CS, and heavily concentrated on providing evidence of impact in terms of education and awareness, climate and environment, and social engagement. A few studies focused on OA, Open Code/Software, and OS general also show some positive (and some negative) societal impacts, but the veracity of societal impact as measured by altmetrics – the majority of the OA literature, is questionable. We are also able to conclude that certain mechanisms and enabling factors lead to societal impact from OS, while certain inhibiting factors get in the way of it.

The results of this study will prove instructive to academic research institutions, funders, publishers, science policymakers, researchers, educators and the general public. There is clear evidence that CS produces a wide variety of beneficial societal impacts, and evidence that signalling OS practices and deploying Open Code/Software in response to societal needs also produces impact. Therefore, investing in these practices is a wise choice for leaders and researchers who wish to foster the societal impact of scientific research. For educators, the evidence that CS fosters learning outcomes and interest in science suggests that the integration of CS within educational settings across age groups is a productive practice. For the general public, in particular people, groups and communities who wish to generate solutions to problems they experience, our findings suggest that CS is a pathway to do so. CS projects and programmes need not be top-down, created by researchers, but can originate at the grassroots and have impact, as our evidence indicates (for example [163,164].

Our findings indicate that additional research is needed to study the societal impact of OS beyond CS, and that more precise and in-depth research is needed to truly establish the societal impact of OA. To date, to our knowledge, wide-scale surveys of the use of OS resources by the general public in nations around the world have not been conducted. Such an approach could provide missing foundational knowledge of which societal actors are using OS resources in which ways and might identify disparities in use that have implications in terms of equity. We also believe that building on large scale quantitative research with in-depth qualitative research with users of OS resources could prove instructive in illuminating causal relationships in OS pathways to impact.

## Data Availability

Datasets and additional materials supporting this article are published on Zenodo, https://zenodo.org/records/10559446 [31]. Supplementary material is available online [242].

## Footnotes

1 PathOS (Open Science Impact Pathways) is a Horizon Europe project aiming to collect concrete evidence of Open Science effects and study the pathways of Open Science practices, from input to output, outcome and impact. https://pathos-project.eu/

2 We did not explicitly include preprints as part of our definition of OA published materials, nor did we specifically include them as a separate category. However, some of the evidence that met our inclusion criteria included discussion of preprints, which we include in the OA subsection of our results.

3 We excluded Open Government Data (OGD) from this study because our focus is on the societal impact of OS practices within academic research. We understand OGD to be data made open by government ministries and offices. In contrast, OFD as we use it refers to data made open by academic researchers. We include here academic research conducted at government funded organisations, like NASA and CERN.

4 Societal impact categories were amended throughout the initial data extraction process as it became clear that they did not adequately capture what we were seeing in the literature. Engagement was amended to ‘social engagement’, participation was removed for redundancy with engagement, education was changed to ‘education and awareness’, and equity, empowerment and privacy/ethics were added as additional categories. The full list of categories used in data charting and data analysis is available as Supplement 3 to this paper.

5 We became aware of SyRF while completing the first phase of this study. After assessing that using it would not alter our methods or results, we decided to implement it for the second phase due to an interest in trialling a dedicated and free-to-use review platform.

6 In this section we present summarised results due to the high volume of literature discussed. Table 3 details all literature described herein with references.

7 Throughout this paper we use the term ‘research’ to refer to academic research rather than ‘science’ to be inclusive of academic disciplines and research fields that may not be considered ‘scientific’. In this section, we use the term ‘science’ because we are reporting trends in the literature, and ‘science’ is the term used in this literature.

8 This is the only paper that demonstrates a causal link between OA and societal impact (as measured by social media engagement). While taking this as a measure of societal impact is debatable (see discussion), we note that all other papers discussed in this section are observational in nature and do not demonstrate causation.

9
https://opendatabarometer.org/

10
https://scikit-learn.org/

11
https://www.ouvrirlascience.fr/the-open-science-monitor/

12
https://open.coki.ac/
